# Shelf Life and Storage Conditions of Universal Adhesives: A Literature Review

**DOI:** 10.3390/polym13162708

**Published:** 2021-08-13

**Authors:** Georgi Iliev, Louis Hardan, Cynthia Kassis, Rim Bourgi, Carlos Enrique Cuevas-Suárez, Monika Lukomska-Szymanska, Davide Mancino, Youssef Haikel, Naji Kharouf

**Affiliations:** 1 Department of Prosthetic Dental Medicine, Faculty of Dental Medicine, Medical University, 1431 Sofia, Bulgaria; g.iliev@fdm.mu-sofia.bg; 2 Department of Restorative Dentistry, School of Dentistry, Saint-Joseph University, Beirut 1107 2180, Lebanon; louis.hardan@usj.edu.lb (L.H.); cynthia.kassis@usj.edu.lb (C.K.); rim.bourgi@net.usj.edu.lb (R.B.); 3 Dental Materials Laboratory, Academic Area of Dentistry, Autonomous University of Hidalgo State, Circuito Ex Hacienda La Concepción S/N, San Agustín Tlaxiaca 42160, Hidalgo, Mexico; cecuevas@uaeh.edu.mx; 4 Department of General Dentistry, Medical University of Lodz, 251 Pomorska St., 92-213 Lodz, Poland; monika.lukomska-szymanska@umed.lodz.pl; 5 Department of Biomaterials and Bioengineering, INSERM UMR_S 1121, Biomaterials and Bioengineering, 67000 Strasbourg, France; endodontiefrancaise@outlook.com (D.M.); youssef.haikel@unistra.fr (Y.H.); 6 Department of Endodontics, Faculty of Dental Medicine, Strasbourg University, 67000 Strasbourg, France

**Keywords:** dentin bonding agents, drug stability, product storage, storage of substances, universal adhesives

## Abstract

This paper presents state of the art universal adhesive systems and the effect of shelf-life and storage conditions on their bond performance. Three topics are explored in this review: an introduction to the topic, the mechanisms responsible for the degradation of the hybrid layer, and the factors that play a role in the stability of universal adhesives. In addition, issues such as potential durability and clinical importance are discussed. Universal adhesive systems are promising but must be handled and stored according to the manufacturer’s instructions, with careful attention given to the details of shelf-life and storage conditions for maximal success. It appears that the components of universal adhesives play an important role in their stability. Furthermore, HEMA-free formulations using methacrylamides lead to longer shelf-life. Further research is needed to prove these hypotheses.

## 1. Introduction

Currently, universal adhesive systems are widely utilized due to their simplified application procedures, the less-sensitive techniques needed, and their more user-friendly nature [1]. Based on this, clinicians are able to use them in either self-etch, etch-and-rinse, and selective enamel-etch modes [2]. However, the storage of the bonding agent and its shelf-life are considered to be key components in the extraoral degradation phenomena of adhesive systems [3]. Accordingly, one should bear in mind that problems related to the preservation of the bonding agent may jeopardize the effectiveness and stability of adhesion to the dental substrate [3,4]. These problems include: difficulty in monomer polymerization, susceptibility to hydrolysis or the degradation of components, and ingredient evaporation [5,6]. In this sense, it is necessary to strictly characterize the recommendations of each company and follow them; otherwise, the deterioration of the adhesive could occur [7]. 

Furthermore, observing the date of expiration should be of great interest [8]. The “shelf-life” of an adhesive refers to the period in which adhesive systems retain an ideal bond [9]. Because the composition of adhesives changes over time, manufacturers always suggest an expiration date after which the adhesive should not be used [10,11]. Nevertheless, dentists do not take into consideration the fact that even as little as 3 months after the expiry date, the clinical efficacy of an adhesive lessens [9]. This is despite the fact that after the opening of an adhesive bottle, the solvents and a small quantity of low molecular weight monomers evaporate, causing the adhesive to become more viscous [12,13]. This chemical alteration leads to poor hybridization and a short adhesive shelf-life [7]. In this case, a uni-dose adhesive package could be recommended [14]. 

Prior to their use in clinical practice, adhesive systems were developed to improve chemical stability against premature polymerization throughout storage [7]. In addition, while numerous degradations can occur due to humidity, light, oxygen, and heat, adhesive systems in their pre-packaged condition should also resist such degradation. Most manufacturers recommend that the storage conditions of adhesives should be between 2 and 25–28 °C prior to the listed expiration date (usually 2 years). However, the transport, shipment, and storage conditions of adhesives before clinical application are not always optimal [12]. Although this clinical reality might strongly influence the end quality of the material, it has rarely been studied in dental biomaterial literature. Therefore, it is of clinical relevance to assess, through a literature review, the effect of shelf-life and storage conditions on the bonding efficiency of universal adhesive systems. In this sense, past studies related to expiry date, shelf-life, and storage conditions will be highlighted.

## 2. Mechanisms Responsible for the Degradation of the Hybrid Layer

The dental adhesion process is based on the creation of a suitable and compact hybrid layer by impregnating the dentin substrate with monomers, regardless of the adhesive layer thickness and the depth of the tags’ penetration into the dentinal tubules [15]. Thus, the hybrid layer is a mixture of collagen, hydroxyapatite, resin monomers, and residual solvents, and its ultimate stability relies on the resistance of the individual components to the degradation phenomena [16]. In general, the more compact and homogeneous the hybrid layer is, the better the stability of the resin–dentin bond will be [17]. The exact mechanisms responsible for the degradation of the hybrid layer can be divided into two major phenomena: the degradation of adhesive resin from the interfibrillar spaces and the degradation and disorganization of collagen fibrils [18]. 

### 2.1. Degradation of Adhesive Resin

Hydrolytic degradation is a chemical reaction that is able to break covalent bonds between polymers, producing a loss of bond strength within the hybrid layer [19]. It is well known that dentin is a moist substrate because of its intrinsic hydrophilic nature [20]. Therefore, contemporary adhesives mix hydrophilic monomers into organic solvents. The hybridization of the adhesive through a dentin substrate is based on the infiltration of such hydrophilic resin monomers into demineralized dentin [21]; whether or not they are polymerized, these hydrophilic monomers can cause high water sorption and generate a hybrid layer that acts as a membrane that permits water movement all along the resin–bond interface [22]. This is followed by the phenomena of hydrolysis and the leaching of resin adhesive: pathways of water-filled channels form along the length of the hybrid layer, which leads to adhesive phase separation from incomplete conversion [23]. 

Accelerated matrix degradation causes the mechanical wear of the exposed adhesive and allows the greater and freer entrance of water and enzymes. This is because of the structure of methacrylate adhesives, which include hydrolytically susceptible groups such as urethane and ester, as well as carboxyl, hydroxyl, and phosphate groups [24]. These monomers can potentially react with the bonding sites inherent to collagen [25] and may be hydrolyzed by enzymatic and chemical degradation in the oral environment [1]. Besides the presence of water, occlusal forces, and acidic chemical agents, the expansion and contraction stresses caused by temperature changes in the oral cavity affect the stability of the resin [26]. The more hydrophilic the adhesive is, the more sensitive it is to this type of degradation [24]. 

It is also known that camphorquinone (CQ), a commonly used photo-initiator, is hydrophobic in nature and causes a suboptimal degree of cure of hydrophilic monomers. An alternative hydrophilic photo-initiator, such as TPO (ethyl 4-dimethylaminobenzoate or diphenyl(2,4,6-trimethylbenzoyl)-phosphine oxide), can be used in addition to CQ in order to drastically improve the degree of cure of hydrophilic monomers used in universal adhesive systems, thus reducing the effect of phase separation [27]. 

### 2.2. Degradation of Collagen Fibrils

The incomplete penetration of the resin into the demineralized dentinal matrix leads to the formation of exposed and water-filled collagen fibrils that are not protected against denaturation problems [1], that can be cleaved by endogenous and exogenous collagenolytic enzymes, and that can be identified immunohistochemically with an enzyme-linked immuno-sorbent assay (ELISA) [21]. 

Host collagenolytic enzymes (matrix metalloproteinases (MMPs) and cysteine cathepsins), also called ‘dentin degradomics’, play a functional role in resin–dentin bond loss both during and after dentin demineralization. Research in this field is being undertaken to suggest new methods for preventing their activity [1,2]. 

This is why, during clinical practices, two important things should be taken into consideration: the shelf-life and the storage conditions of universal adhesives.

## 3. Factors That Play a Role in the Stability of Universal Adhesives 

A number of concerns have been raised concerning the bonding effectiveness of multimode adhesives, mainly in terms of stability, although this tends to be very depending on the material [1,28]. Unfortunately, hydrolysis phenomena, the evaporation of adhesive ingredients, the repeated opening of the adhesive bottle, and excessive storage time may impair bond strength and increase both nanoleakage expression (NL) and hybrid layer permeability [7,9,29]. Indeed, these factors can have an impact on the shelf-life stability of a material specifically if it is used after the date of expiry suggested by the manufacturer [9]. Bonding agents use chemicals that significantly weaken with time, especially when they are subject to elevated temperatures [30]. However, the main aim is that adhesion to tooth substrates should preferably stay stable over time, since the success of the restorative procedure relies on this outcome [1]. This may be possible if environmental conditions such as temperature and humidity are controlled in an adequate manner, preventing the phase separation of the adhesive formulation. As a consequence, this may prevent the potential loss of bonding ability of the adhesive [31]. 

**Figure 1:**
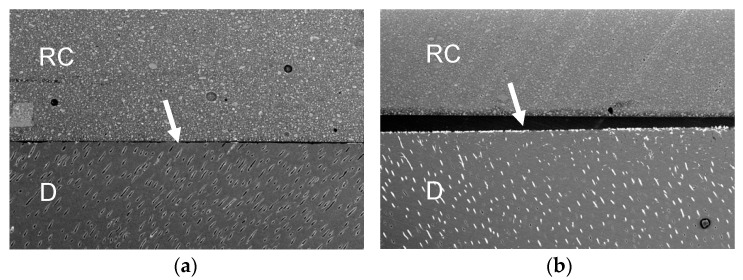
Representative backscatter SEM images of the resin–dentin adhesive interfaces of SBU after 24 h. (**a**) Received by the manufacturer; (**b**) expired. RC, resin composite. D, dentin. White arrows indicate adhesive layer; 250x.

**Figure 2:**
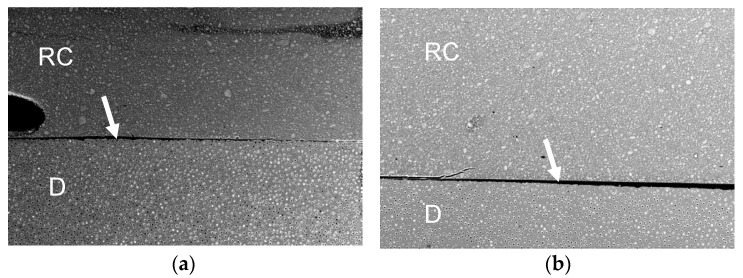
Backscatter SEM images of the resin–dentin adhesive interfaces of PBE after 24 h. (**a**) Received by the manufacturer; (**b**) expired. RC, resin composite. D, dentin. White arrows indicate adhesive layer; 250x.

**Figure 3:**
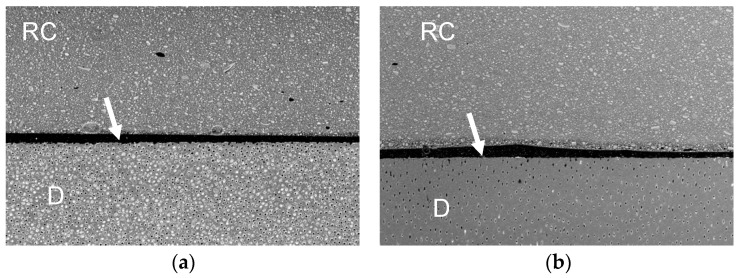
Backscatter SEM images of the resin–dentin adhesive interfaces of OBU after 24 h. (**a**) Received by the manufacturer; (**b**) expired. RC, resin composite. D, dentin. White arrows indicate adhesive layer; 250x.

**Figure 4:**
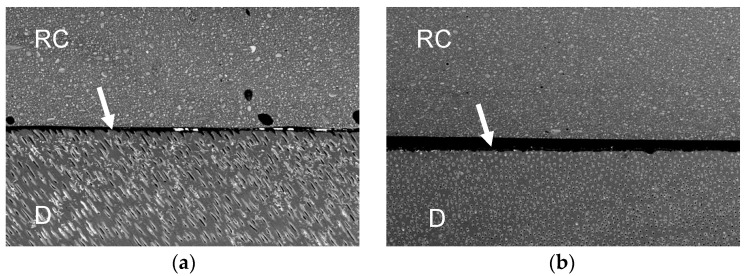
Backscatter SEM images of the resin–dentin adhesive interfaces of OCU after 24 h. (**a**) Received by the manufacturer; (**b**) expired. RC, resin composite. D, dentin. White arrows indicate adhesive layer; 250x.

Figure 1, Figure 2, Figure 3 and Figure 4 represent the scanning electron microscopy (SEM) in backscattered mode of four universal adhesive systems (one is as received by the manufacturer, -a-, and the other is expired, -b-). In these images, nanoleakage is represented by ion silver deposition within the adhesive layer. Nanoleakage is used as an indirect method to evaluate the quality of resin–dentin bonds and this feature represents the location of defects within the adhesive layer that might indicate some kind of degradation. Figure 1 shows a Single Bond Universal, or SBU (3M ESPE, St. Paul, MN, USA), adhesive with an increase in NL after the date of expiration. SBU is a 2-hydroxyethyl methacrylate (HEMA)-containing adhesive, and the presence of HEMA has been related to the inhibition of the nanolayering chemical bonding of the 10-methacryloyloxydecyl dihydrogen phosphate (10-MDP) monomer, which could increase nanoleakage [32]. Figure 2 shows the Prime&Bond Elect, P&B (Dentsply Caulk, Milford, DE, USA), adhesive, which maintains almost the same NL after the date of expiration. This adhesive is formulated without HEMA; the absence of this monomer could probably promote the better cross-linking density of the adhesive layer, making it less prone to degradation [33]. Furthermore, Figure 3 shows the OptiBond Universal, OBU (Kerr, Orange, CA, USA) adhesive, while Figure 4 shows OneCoat Universal, OCU (Coltène/Whaledent Inc., Cuyahoga Falls, OH, USA). For these adhesives, no substantial increase in the nanoleakage was observed after the expiration date. It is possible that the pH of these materials, considered to be mild, could be helpful in preventing the degradation of their components.

The adhesive–collagen complex comprises extremely dissimilar structures and a heterogeneous composition. Examination of the diverse interfaces of these hybrids presents several analytical challenges. Therefore, in situ characterization is crucial to recognizing key factors in designing these adhesives.

It is important to mention that the degree of conversion (DC) is a feature that is generally influenced by the concentration and type of photoinitiator used. Though it was not stated in several of the safety data sheets, most of the bonding reviewed in this study was based on the CQ photoinitiation system [8]. 

Several studies have proven that, in acidic environments, the stability and effectiveness of this photoinitiator (CQ) were low [34,35]. This could be explained by the fact that an acid–base reaction appears between the acidic monomers and the amines, thus preventing the amine from proceeding as a polymerization co-initiator [36]. In addition, the interaction between the acidic monomer and the amine might neutralize the acidic functional monomer, worsening its capacity to form stable bonds with the hydroxyapatite of the dentin substrate [34].

Furthermore, Raman microspectroscopy has evolved to become a versatile method for the in situ structural characterization of adhesive-dentin interfaces [2,37]. The Raman spectrum can probe the chemical structure and offer a clear “fingerprint” of the molecules present in a sample. It can be used for both quantitative determination and qualitative identification [38]. To analyze the adhesive–collagen hybrids before and after the date of expiration, micro-Raman spectroscopy could be the solution for future studies. Additionally, one should bear in mind that other methods, such as Fourier transform infrared (FTIR) chemical imaging, can be useful for examining the molecular chemical features of the adhesive–dentin interfaces. The relative chemical composition and homogeneity across the length and breadth of the adhesive–dentin interfaces and the degree of cure can be determined [39,40].

In this regard, as non-ideal storage conditions of universal adhesives can degrade some of the components and decrease the bonding ability of the material, a better understanding of the expiry date recommended by the manufacturer and better storage conditions could help in finding solutions that aid in enhancing the bond performance of universal adhesives. 

**Figure 5:**
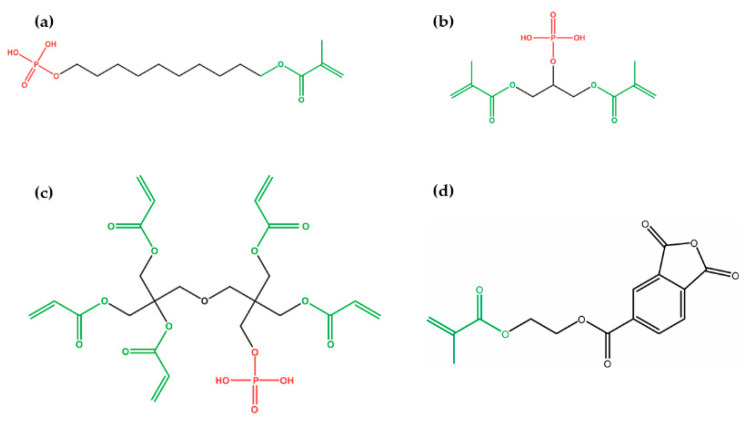
Chemical structure of the acidic monomers used in some universal adhesive systems: (**a**) 10-methacryloyloxydecyl dihydrogen phosphate; (**b**) glycerol phosphate dimethacrylate; (**c**) dipentaerythritol penta-acrylate phosphate; (**d**) 4-methacryloxyethyl trimellitic anhydride.

In 2014, Cardoso et al. evaluated the effect of shelf-life simulation (storage cycle in climate chamber at 40 °C with 50% relative humidity) on the long-term dentin bond strength of Single Bond Universal (3 M ESPE, St. Paul, MN, USA) used in self-etch mode [12]. Interestingly, this universal adhesive produced a satisfactory hybrid layer which strengthened the bond after 4 weeks of shelf-life simulation or even after 6 months of storage in water when compared to 8 and 12 weeks of shelf-life simulation. This could be explained by the fact that this adhesive has a specific formulation which is different from other adhesives. The formulation includes Vitrebond copolymer, which is famous for its promotion of higher degrees of stability against external factors such as humidity, which is a cause of bond deterioration [41]. Additionally, the temperature and humidity were specified in this study, which resulted in better monomer reticulation with this polyalkenoic acid copolymer. Another monomer found in the formulation is MDP, which is a functional monomer that enhances the hybrid layer stability, thus resulting in long-term bond effectiveness [42]. When 10-MDP is mixed with HEMA (Figure 5), the wettability of dentin substrate is improved, leading to a stronger interaction between this adhesive and hydroxyapatite [12].

However, another study counters these claims by demonstrating an increase in nanoleakage as a result of the degradation of MDP-containing primers when stored at a temperature of 40 °C [43]. The temperature and humidity were decided in the shelf-life simulation. This does not reflect the actual parameters that are dictated by the environment within the import/export stages of the adhesive [12]. Fluctuations, naturally, should cause significant changes in the physical, mechanical, and chemical properties of adhesives, as mentioned by previous study [31]. In order to obtain valuable information about the degradability of universal adhesives, manufacturers should provide a standard process during the manufacturing of new adhesive systems by performing shelf-life simulations [12]. 

In 2014, Pongprueksa et al. studied the effect of evaporation on the shelf-life of a one-bottle universal adhesive: Scotchbond Universal (SBU, 3M ESPE, Seefeld, Germany) [7]. It is well known that the wetting behavior of adhesives is improved by adding different hydrophilic monomers and solvents [16]. Organic solvents serve different purposes, such as removing the water that exists between the collagen fibers and dissolving the amphiphilic resin, thus increasing the surface tension and facilitating the penetration of the adhesive into the substrate [27]. The main solvents used in adhesive systems include acetone, ethanol, and water (evaporation time between solvents varies) [24]. It is obvious that the amount of solvent in an adhesive can differ between the first and last use due to the evaporation of solvents [7]. The occasional opening of the bottle may jeopardize the shelf- life of the universal adhesive system by impairing the bond strength and reducing the DC [9]. However, these negative effects were realized only after more than 50% evaporation [7]. Moreover, Perdigao et al. studied the effects of the repeated opening of a two-step etch-and-rinse adhesive on bond strength. They intentionally left the bottle open for one minute, two times a day, for three weeks and evaluated the bond strength. They found a weakened bond strength after three weeks for the acetone-containing adhesive and concluded that solvent evaporation may decrease the shelf-life of adhesives [44]. Thus, it is unlikely that the shelf-life in a clinical condition will be impaired by evaporation, especially when the adhesive is used in accordance with the manufacturer’s recommendations [7]. 

In addition, shelf-life can be an issue when an acidic monomer and HEMA are used in an adhesive formulation, as they tend to degrade over time in the presence of solvents and water [12]. Furthermore, the type of solvent plays an important role in the bonding stability [6,12]. In 2019, Cuevas-Suárez studied the impact of shelf-life on the bonding performance of eight adhesives. Of these eight, the following five universal adhesives were evaluated: Single Bond Universal, SBU (3M ESPE, St. Paul, MN, USA); Tetric Bond Universal, TBU (Ivoclar Vivadent, Schaan, Liechtenstein); OneCoat Universal, OCU (Coltène/Whaledent Inc., Cuyahoga Falls, OH, USA); OptiBond Universal, OBU (Kerr, Orange, CA, USA); and Prime&Bond Elect, P&B (Dentsply Caulk, Milford, DE, USA). Microtensile bond strength (μTBS) in SBU, OCU, and OBU adhesives decreased correspondingly following evaluation at the halfway point and end of their shelf-life, while the other adhesives, TBU and P&B, remained stable during all the shelf-life evaluated [8]. Hence, shelf-life simulation should be a routine practice for the evaluation of universal adhesives [45]. 

The decrease in bond strength might be linked to the chemical formulation of these materials. Based on the manufacturer’s safety data sheet, SBU materials are formulated using 10-MDP, whereas OBU contains glycerol phosphate dimethacrylate (GDMA-P) (Figure 5). In contrast, although this was not stated for OCU, one of the monomers mentioned above was probably used in its composition. Previous data show that ester-based adhesive formulations with an acidic pH are very susceptible to hydrolysis [46,47]. As a consequence, ethylene glycol, free methacrylic acid, other alcohol derivatives, and free phosphoric acid are obtained. This hydrolytic phenomenon alters the chemical formulation of the universal adhesive throughout its storage in the warehouse or dental office, changing its properties and hindering the bond strength between substrates [45].

Remarkably, the P&B and TBU adhesives maintained bond strength values after the shelf-life simulation period. The universal adhesive P&B contains a dipentaerythritol penta-acrylate phosphate monomer (PENTA-P, Figure 5) in its formulation [8]. Although the degradation mechanism of this monomer is still unknown, it could be hypothesized that, unlike the functional monomer 10-MDP, the availability of five vinyl groups in PENTA-P compared to one in 10-MDP within its chemical structure could make it even more resistant to the hydrolytic degradation phenomenon. Consequently, when hydrolysis takes place and breaks a vinyl group off the main structure of the monomer, four vinyl groups remain available to maintain a connection to the phosphate group, which permits copolymerization with the other monomers [48]. With regard to TBU adhesive, with a relatively high pH of about 3 [49], it could be that the degradation rate of the methacrylated phosphoric acid ester where this material is based is slower than it is in the other materials. Because the hydrolysis of ester bonds into acidic aqueous media is dependent on how acidic the materials are, the use of self-etch adhesives with relatively high pH could help in obtaining materials with high shelf-life stability [8]. Although it was not mentioned in some of the safety data sheets, most of the materials used in the current study were based on the CQ/EDAB photoinitiation system. A previous study has shown that, under acidic conditions, the stability of this photoinitiator system was low [50]. An acid–base reaction occurs between the acidic monomers and the amines, preventing the amine from assuming the role of the polymerization coinitiator [51]. The interaction between the amine and the acidic monomer, on the other hand, might neutralize the acidic functional monomer, weakening its capacity to produce stable bonds with the hydroxyapatite of the dentin [50]. The reduction in the bond strength values after the shelf-life simulation observed in this study could also have occurred due to this neutralization process. Additionally, the findings stated that humidity could also affect the shelf-life stability of dental adhesives [8]. The manufacturers contribute to the issue by not providing the range of humidity in which the materials should be stored. Future research should therefore be undertaken to find the effect of this variable on the degradation rate of the components where these materials are based. 

In 2020, Mazzitelli et al. evaluated the iBond Universal (Heraeus Kulzer, GmbH, Hanau, Germany) in self-etch mode as received by the manufacturer or three months after expiry. The μTBS, the NL, and the endogenous enzymatic activity were studied. The study showed a decrease in the bond performance of the universal adhesive tested beyond the expiry date. Further, the increase in the activity of the endogenous enzymes recorded after the expiration of the adhesive may lead to the weakened durability of the restorations. These results could be an effect of the universal adhesive formulation [9]. Based on what was stated, iBond universal is an adhesive based on acetone, urethane dimethacrylate (UDMA), and 4-methacryloxyethyl trimellitate anhydride (4-META). Acetone is highly volatile and allows the rapid evaporation of the adhesive components [52], thus participating in obtaining strong adhesion forces. However, during the drying process, incomplete solvent volatilization may affect the degree of polymerization of the adhesive [53]. A previous report has shown that ester-based adhesive compositions with an acidic pH are susceptible to hydrolytic degradation [46]. Once an ester cleavage occurs, the 4-META monomer is hydrolyzed to establish 4-MET (4-methecryloxyethyl trimellitic acid). This phenomenon is likely to negatively influence the efficiency of the polymerization and the infiltration phase of the demineralized substrates [54], leading to premature monomer degradation [8]. The longevity of the bond could consequently be hindered [55]. 

In addition, in situ zymography was executed in the current study, and the results attained indicate that the substrate can be highly degradable. The universal adhesive used after the expiry date creates an interface between the adhesive and the dentin which possesses the higher collagenolytic activity responsible for the degradation of bond strength with time [9,56]. Therefore, the universal adhesive that is acclaimed by dentists worldwide should only be used within the range provided by the manufacturer.

## 4. Final Considerations 

Universal adhesives contain chemicals that may suffer hydrolytic degradation due to inadequate storing conditions and long storage times. This characteristic may weaken the bond ability of these materials over time. In addition, the frequent opening of the bottle can evaporate the solvent found inside them, decreasing the shelf-life. In this sense, storing universal adhesives in a refrigerator and recapping the bottle of the adhesive after use are recommended techniques that could decrease the degradation rate of the materials.

On the other hand, it seems that the components of universal adhesives play an important role in their stability. Furthermore, HEMA-free formulations using methacrylamides lead to longer shelf-life. Further research is needed to prove these hypotheses. 

## Data Availability

The data presented in this study are available on request from the corresponding author.
